# Soy isoflavone reduces LPS-induced acute lung injury via increasing aquaporin 1 and aquaporin 5 in rats

**DOI:** 10.1515/biol-2022-0560

**Published:** 2023-02-10

**Authors:** Xiaobo Wang, Yili Zhang, Xiuyun Zhou, Xiumei Xia, Weijun Teng, Lin Sheng, Jing Ding

**Affiliations:** Department of Gastroenterology, Affiliated Jinhua Hospital, Zhejiang University School of Medicine, Jinhua, Zhejiang, 321000, China; Department of Health Management Center, Affiliated Jinhua Hospital, Zhejiang University School of Medicine, Jinhua, Zhejiang, 321000, China; Department of Blood Purification Center, Affiliated Jinhua Hospital, Zhejiang University School of Medicine, Jinhua, Zhejiang, 321000, China; Department of Imaging Medicine, Affiliated Jinhua Hospital, Zhejiang University School of Medicine, Jinhua, Zhejiang, 321000, China; Department of Respiratory Medicine, Affiliated Jinhua Hospital, Zhejiang University School of Medicine, Jinhua, Zhejiang, 321000, China

**Keywords:** soy isoflavone, aquaporin 1, aquaporin 5, acute lung injury, inflammation

## Abstract

Acute lung injury (ALI) followed with severe inflammation and oxidative stress. Anti-inflammatory and antioxidant are the properties of aquaporin 1 (AQP1) and aquaporin 5 (AQP5). The goal of this study was to see if soy isoflavone can diminish lipopolysaccharide (LPS)-induced ALI and the underling mechanism. LPS-induced ALI was given to Sprague–Dawley rats 14 days following oophorectomy. One hour before the LPS challenge, estradiol (1 mg/kg) was administered subcutaneously as positive control and soy isoflavone was intragastric administration for 14 days prior to LPS challenge with different doses. Six hours after LPS challenge, the pulmonary edema, pathophysiology, inflammation, and the oxidative stress in lung tissues of rats were discovered. We found that soy isoflavone can reduce pulmonary edema and the lung pathology in a dose-dependent manner. Furthermore, tumor necrosis factor-alpha, interleukin-1β, and interleukin-6 were decreased in rats treated with soy isoflavone. Meanwhile, soy isoflavone reduced pulmonary oxidative stress by decreasing malondialdehyde levels, while increasing superoxide dismutase levels in lung tissues in a dose-dependent manner. Mechanically, we found that the mRNA and protein level of AQP1 and AOP5 were increased in lung tissues of rats treated with soy isoflavone compared the LPS-treated rats. Thus, soy isoflavone alleviates LPS-induced ALI through inducing AQP1 and AQP5.

## Graphical abstract

**Figure:**
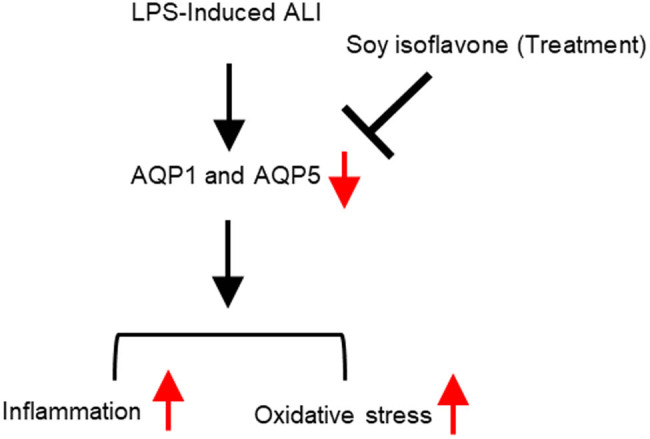

## 1 Introduction

Acute inflammatory disease associated to oxidative stress is caused by sepsis-induced acute lung injury (ALI) that produces significant morbidity and death [1–3], posing a serious health risk. As a result, finding new mechanisms and therapies for ALI remains difficult.

More and more studies show that natural products possess general health-promoting benefits to human diseases. Amin et al. found that saffron and its main components have an anti-proliferation effect on colon cancer cells [4]. The antioxidant effects of natural products play an important role in treating diseases. Vitamin C and Vitamin E are beneficial to diabetic rats by improving biochemical parameters [5,6]. Amin et al. also found that spirulina has a protective effect on cadmium-induced hepatotoxicity in rats through antioxidant activity [7] and chlorella is good for diabetic rats by restoring the function of pancreatic insulin-secreting cells [8]. Aescinate saponins and diaescinate saponins have protective effects on liver injury in rats [9]. Several studies have shown that soy isoflavone has anti-inflammatory and antioxidation properties that help to regulate the progression of illnesses such as cancer [10], cardiovascular diseases [11], diabetes [12,13], and cerebrovascular disease [14]. To protect against diabetic nephropathy, soy isoflavone can lower the cytokinesis of interleukin-1 (IL-1), interleukin-6 (IL-6), and nicotinamide adenine dinucleotide phosphate (NADPH) oxidase gene expression [12]. The activities of soy isoflavone in ALI, on the other hand, are mostly unknown.

Aquaporins (AQPs) are one of the respiratory system's cell membrane transporters, with AQP1 and AQP5 being the most important [15]. AQPs in lung tissues have been found to eliminate excess fluid from the alveolar space in studies [16,17]. Furthermore, AQP1 and AQP5 are involved in the development of pulmonary edema induced by ALI, hyperoxia, hemorrhagic shock, acute renal injury, and acute pancreatitis [18,19]. After lipopolysaccharide (LPS)-induced diffuse vascularization, AQP5 expression was reduced in pulmonary edema [20,21]. In mice, knocking out AQP1 in the lungs worsened pulmonary edema [20,22]. However, it is unclear if soy isoflavone’s anti-inflammatory and antioxidant effects are mediated through AQPs.

We show that soy isoflavone can decrease oxidative stress and inflammation in a rat model of LPS-induced ALI via dose-dependently activating AQP1 and AQP5. As a result, soy isoflavone may be a new treatment for ALI.

## 2 Methods

### 2.1 Drugs

Soy isoflavone was acquired from Shanghai Yuanye Co. Ltd (B25058) and dissolved in dimethyl sulfoxide (sigma, D2650). Estradiol was purchased from Sigma (USA, E8875) and dissolved in ethanol. LPS was purchased from Sigma-Aldrich (USA), which was derived from *Escherichia coli* O111: B4, and dissolved in saline (Gibco, Grand Island, NY, USA).

### 2.2 Animals

The National Institutes of Health produced the Guide for the Care and Use of Laboratory Animals, which outlined how animal research should be conducted (National Institutes of Health publication No. 85-23, revised 1985). The Hangzhou Hibio Technology Co. Institutional Ltd Animal Care and Use Committee approved the procedure (IACUC protocol number: HBFM3.68-2015). Shanghai SLAC Laboratory Animal Co. Ltd provided Sprague–Dawley rats (female, oestrum) [23]. Rats were kept in a pathogen-free environment with a 12 h light–dark cycle in the same room at 18–24°C and 40–70% humidity. All rats were treated in accordance with the Institutional Animal Care guidelines.


**Ethical approval:** The research related to animal use has been complied with all the relevant national regulations and institutional policies for the care and use of animals and has been approved by The Hangzhou Hibio Technology Co. Institutional Ltd Animal Care and Use Committee (IACUC protocol number: HBFM3.68-2015).

### 2.3 LPS-induced ALI and soy isoflavone or estrogen preconditioning

Oophorectomy was performed on Sprague–Dawley rats (*n* = 60) as reported in previous studies [24–26]. After removing the hair and disinfecting the surgical site, ovariectomy was performed through bilateral incisions in the skin and small bilateral sections via the muscle layer at the angle between the last rib and vertebral column. The skin was incised together with the dorsal muscles and the peritoneal cavity was accessed. The ovary was identified, surrounded by a variable amount of fat. After vascular ligation, the connection between the fallopian tube and the uterine horn was cut and the ovary was removed. Rats were randomly assigned to one of six groups (*n* = 10) 14 days after oophorectomy. OVXC: ovariectomy without LPS as a control group; OVXM: ovariectomy with LPS-induced ALI group; L: ovariectomy with LPS-induced ALI and pre-treated with low dose soy isoflavone (5 mg/kg) group; M: ovariectomy with LPS-induced ALI and preprocessed with medium dose soy isoflavone (10 mg/kg) group; H: ovariectomy with LPS-induced ALI and high dosage soy isoflavone (20 mg/kg) preprocessing group; E: ovariectomy with LPS-induced ALI and with estradiol (1 mg/kg) as positive control group. LPS (10 mg/kg) was injected intraperitoneally in rats to induce ALI, and the animals were anaesthetized with ketamine (50 mg/kg) and xylazine (50 mg/kg) intraperitoneally to be fully anaesthetized and sacrificed after 6 h. The goal of this study was to see if soy isoflavone and estradiol may help with LPS-induced ALI. Estradiol (1 mg/kg) was given subcutaneously for 1 h before LPS management, and soy isoflavone was administered intragastrically for 14 days prior to LPS management. The protocol was permitted by the Animal Experimentation Ethics Committee.

### 2.4 Lung tissue wet/dry weight ratio

We used the methods in ref. [27] to identify lung edema in rats following LPS-induced ALI, collecting right lung tissues (*n* = 4) and removing surface blood. The wet weight refers to the weight of the samples, whereas the dry weight refers to the weight of tissue that has been dried at 60°C for 48 h. Each individual rat lung’s pulmonary edema is represented by a wet/dry weight ratio.

### 2.5 Permeability of lung

The animals were all injected with Evans blue (50 mg/kg) through femoral vein 15 min before the animals were killed. The lungs were removed after thoracectomy, and the surrounding tissues were cut off from the upper lobe of the right lung. The lungs were immersed in formamide solution (20 mg/100 g animal weight) and placed in a temperature chamber of 45–50°C for 72 h, until all the pigments in the tissues were leached, then the tissues were removed and centrifuged. Using spectrophotometer to detect the supernatant at 620 nm with 96-well plate. Optical density value of formamide solution was determined. Evans blue content per gram of wet lung tissue was calculated according to the concentration corresponding to the standard curve.

### 2.6 Total protein concentration of bronchial lavage fluid (BALF) and cell counts

We followed the methods [27] after 6 h of LPS treatment, BALF samples were obtained (*n* = 6) by flushing the lungs with 1 mL of 1× PBS (Gibco, Grand Island, NY, USA), and the fluid was centrifuged at 1500 rpm for 5 min at 4°C, the total protein concentration of BALF in supernatant was determined using a BCA protein quantification kit. The sedimented cell pellets were re-suspended in 0.5 mL PBS, then cells were counted using a hemocytometer and Wright-Giemsa staining with a light microscope.

### 2.7 Histology and immunohistochemistry

Pathological alterations in left lung morphology (*n* = 4) were assessed in methyl Carnoy's fixed, paraffin-embedded tissue slices (4 μm) with hematoxylin and eosin (HE) staining, according to the methods in ref. [28]. A microwave-based antigen retrieval approach was used to execute immunohistochemistry in paraffin slices. This investigation employed antibodies against AQP1 (Abcam, ab65837) and AQP5 (Abcam, ab78486). Positive cells were counted using a 0.0625 mm^2^ graticule placed in the eyepiece of the microscope and represented as cells per millimeter square (cells/mm^2^) or were quantitatively scored using Media Cybernetics’ Image-Pro plus software (Bethesda, USA) as stated in ref. [29].

### 2.8 Electron microscopy

Fractions of the left lung tissues (*n* = 4) were pre-fixed in a solution of 2.5% glutaraldehyde and 1% osmium tetroxide, post-fixed in 1% OsO_4_, dehydrated in an escalating sequence of alcohols, and embedded in epoxy resin, as reported before [30]. Uranyl acetate and lead citrate were used to stain ultrathin sections. A transmission electron microscope (HITACHI H-600, Japan) was used to detect the samples.

### 2.9 Real-time PCR

Total RNA from lung tissues (*n* = 6) was isolated using the RNeasy Isolation Kit (Qiagen, Valencia, CA) according to the manufacturer’s instructions. Bio-Rad iQ SYBR Green supermix and the Opticon2 were used for real-time PCR (Bio-Rad, Hercules, CA). Primers for AQP1 are as follows: forward 5′−TCACTTGGCCGAAATGACCTG-3′ and reverse 5′−GTCCCACCCAGAAAATCCAGT−3′. Primers for AQP5 are as follows: forward 5′−TCCAGGACCACACCAGAAAG−3′ and reverse 5′−ATAAAATAGCACTCCGTGAGCC-3′. Primers for β-actin are as follows: forward 5′−ACTGCCGCATCCTCTTCCTC−3′ and reverse 5′−GAACCGCTCATFGCCGATAGTG−3′. Reaction specificity was confirmed by melting curve analysis. As previously disclosed [31,32], the ratios for the AQP1 and AQP5 mRNA were normalized with β-actin and represented as mean ± SEM.

### 2.10 Western blot

The protein of lung tissues (*n* = 6) was extracted using RIPA lysis buffer, and western blot analysis was done as reported previously [32]. After blocking with 5% skim milk, membranes were incubated overnight at 4°C with 1:1,000 dilutions of primary antibodies against AQP1 (Sigma, AB2219), AQP5 (Sigma, AB15858), and GAPDH (Abcam, ab8245). Membranes were incubated with goat anti-rabbit (Amyjet Scientific, 111-035-003) for 1 h at room temperature the next day after being rinsed with 1× TBST 5 min for three times. Blots were examined using a chemiluminescent substrate and molecular band intensity was determined by densitometry after being washed with 1× TBST 5 min for three times.

### 2.11 ELISA

Tumor necrosis factor-alpha (TNF-α; mlbio, ml002859), IL-1 (mlbio, ml003060), and IL-6 (mlbio, ml102828) levels were measured in supernatant of BALF (*n* = 6) using Quantikine ELISA kits according to the product procedures.

### 2.12 Determination of malondialdehyde (MDA) and superoxide dismutase (SOD)

Using saline to extract supernatant of lung tissues (*n* = 6) and analysis was performed following the MDA (Beyotime, S0131) and SOD (Abcam, ab65354) kits’ instruction. 

### 2.13 Statistical analyses

The results of this investigation were given as mean ± SEM. GraphPad Prism 6.0 was used to conduct statistical analyses, which included one-way ANOVA and a Newman–Keuls post-test (Graph Pad Software, San Diego, CA, USA).

## 3 Results

### 3.1 LPS-induced acute pulmonary edema is protected by soy isoflavone in a dose-dependent manner

**Figure 1:**
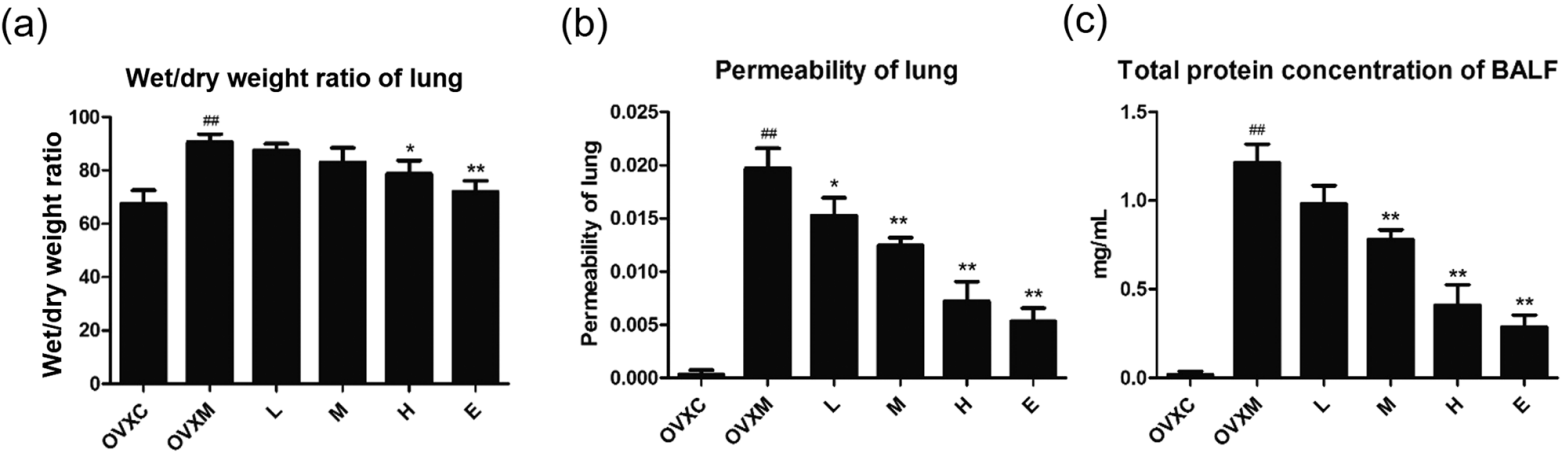
Soy isoflavone reduces the wet/dry weight ratio, lung permeability, and total protein concentration of BALF in a dose-dependent manner. (a) Quantitative study of wet/dry weight ratio of lung, *n* = 4. (b) Quantitative analysis of lung permeability, *n* = 4. (c) Quantitative analysis of total protein concentration of BALF, *n* = 6. Data represent mean ± SEM for groups of 4–6 rats (^##^ *P* < 0.01 vs OVXC group; ^*^ *P* < 0.05, ^**^ *P* < 0.01 vs OVXM group).

The basic pathogenic mechanism of ALI is acute pulmonary edema. To investigate the effects of soy isoflavone on LPS-induced pulmonary edema, we used Sprague–Dawley rats 14 days after oophorectomy in a LPS-induced ALI paradigm. The preventive effects of soy isoflavone on LPS-induced ALI were next investigated. A wet-to-dry weight ratio, lung permeability, and BALF total protein content were used to assess pulmonary edema. LPS treatment resulted in significant pulmonary edema in rats, with increased wet-to-dry weight ratio, lung permeability, and BALF total protein content. Pre-treatment with soy isoflavone reduced LPS-induced pulmonary edema compared to the rats in OVXM group in a dose-dependent manner. Furthermore, the high-dose soy isoflavone had the same impact as the estradiol pre-treatment (Figure 1).

### 3.2 LPS-induced ALI is protected by soy isoflavone in a dose-dependent manner

**Figure 2:**
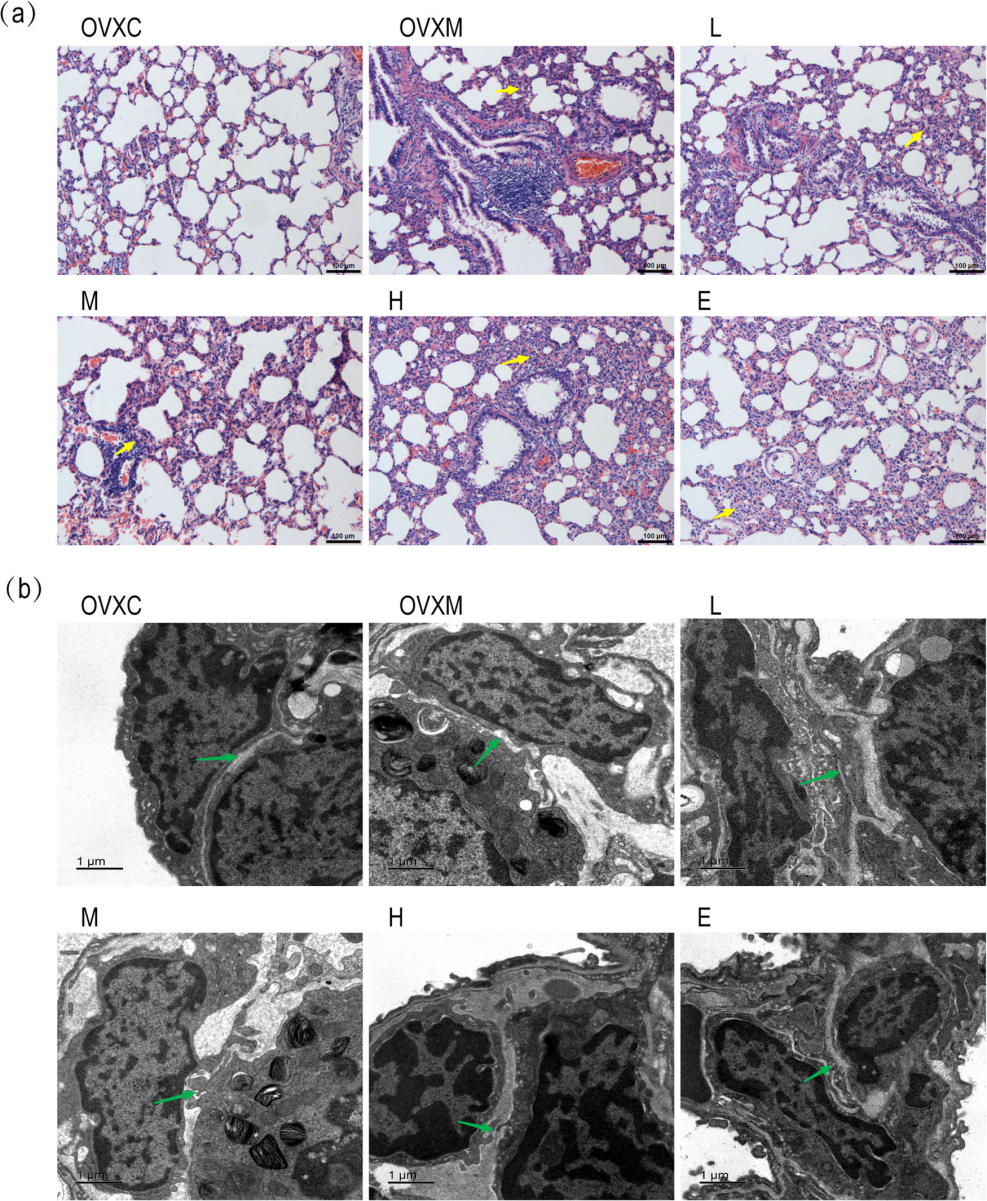
Soy isoflavone reduces the pathological changes of lung tissues. (a) Representative HE staining pictures of lung tissues 6 h after LPS injection in rats, scale bar = 100 μm, the yellow arrow shows the injured site of lung tissue. (b) Electron microscope pictures of lung ultrastructure taken 6 h after LPS treatment, scale bar = 1 μm, and the green arrow shows the intercellular space and swallowing vesicles in the cytoplasm of lung tissues. Data represent mean ± SEM for groups of four rats.

Lung histology was assessed using HE staining to investigate the effects of soy isoflavone on LPS-induced ALI. In rats, LPS treatment resulted in severe lung damage, including edema, congestion, and thickening of the pulmonary septum. Pre-treatment with soy isoflavone decreased LPS-induced lung damage more than the rats in the OVXM group in a dose-dependent manner. Furthermore, high-dose soy isoflavone had the same effect as pre-treatment with estrogen (Figure 2a).

Furthermore, electron microscopy of lung sections was used to examine the ultrastructure of the lungs. In rats, LPS treatment resulted in increased intercellular space and swallowing vesicles in the cytoplasm of lung tissues. When compared to the rats in the OVXM group, pre-treatment with soy isoflavone reduced LPS-induced intercellular space and decreased swallowing vesicles in the cytoplasm of lung tissues in a dose-dependent manner. Furthermore, the high-dose soy isoflavone had the same impact as the estradiol pre-treatment (Figure 2b).

### 3.3 Soy isoflavone has an anti-inflammatory action that is dose-dependent and protects against LPS-induced ALI

In addition, the anti-inflammatory effect of soy isoflavone on LPS-induced lung inflammation was investigated. We discovered that administering LPS to rats resulted in severe lung inflammation with a mass of inflammatory cell infiltration in the BALF. At 6 h after LPS administration, BALF was assessed. After LPS treatment in rats, the amount of white blood cells, neutrophils, lymphocytes, and monocytes increased in BALF. Pre-treatment with soy isoflavones, on the other hand, reduced this rise in a dose-dependent manner. Furthermore, the high-dose soy isoflavone had the same impact as the estradiol pre-treatment (Table 1).

**Table 1:** Soy isoflavone attenuates inflammatory cells of BALF in rats

	White blood cells (10^9^/L)	Neutrophils (10^9^/L)	Eosinophils (10^9^/L)	Lymphocytes (10^9^/L)	Monocytes (10^9^/L)	Basophilic granulocytes (10^9^/L)
OVXC	0.51 ± 0.07	0.28 ± 0.06	0.00 ± 0.00	0.11 ± 0.02	0.11 ± 0.01	0.00 ± 0.01
OVXM	1.47 ± 0.05	0.89 ± 0.09	0.01 ± 0.01	0.24 ± 0.06	0.34 ± 0.02	0.00 ± 0.01
L	1.24 ± 0.09	0.72 ± 0.10	0.01 ± 0.01	0.22 ± 0.03	0.28 ± 0.02	0.01 ± 0.01
M	1.06 ± 0.02	0.64 ± 0.02	0.01 ± 0.02	0.18 ± 0.01	0.21 ± 0.03	0.01 ± 0.02
H	0.74 ± 0.04	0.42 ± 0.02	0.00 ± 0.00	0.14 ± 0.01	0.17 ± 0.03	0.00 ± 0.01
E	0.82 ± 0.07	0.54 ± 0.05	0.00 ± 0.00	0.13 ± 0.02	0.15 ± 0.04	0.01 ± 0.01

OVXC: ovariectomy without LPS as a control group; OVXM: ovariectomy with LPS-induced ALI group; L: ovariectomy with LPS-induced ALI and pre-treatment with low dose soy isoflavone (5 mg/kg) group; M: ovariectomy with LPS-induced ALI and pre-treatment with medium dose soy isoflavone (10 mg/kg) group; H: ovariectomy with LPS-induced ALI and pre-treatment with high dose soy isoflavone (20 mg/kg) group; E: ovariectomy with LPS-induced ALI and pre-treatment with estrogen (1 mg/kg) as positive control group. Data represent mean ± SEM for groups of six rats.

**Figure 3:**
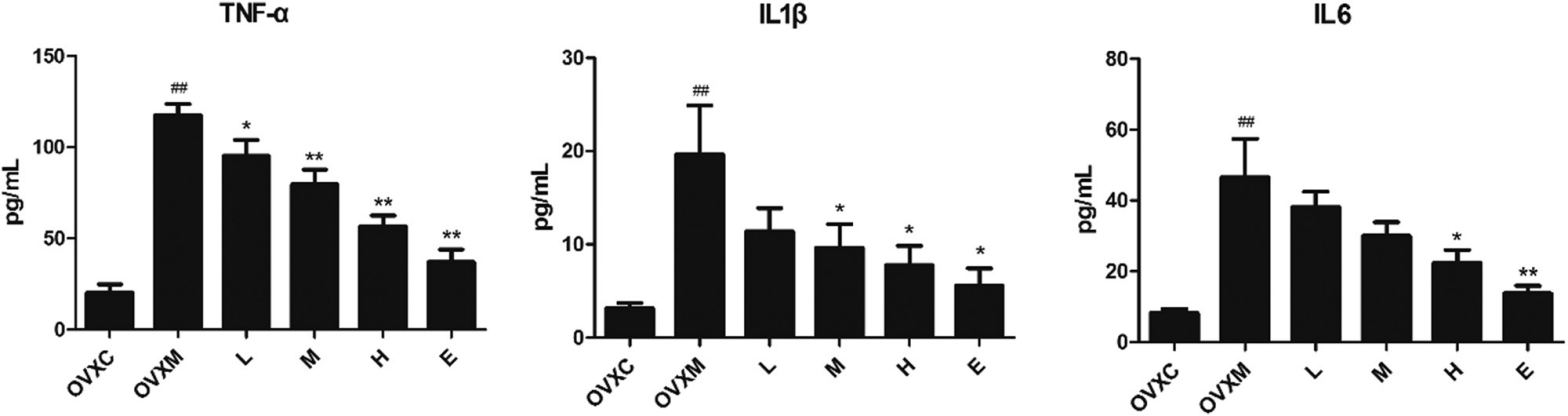
Soy isoflavone dose-dependently decreases the TNF-α, IL-1β, and IL-6 of BALF. The quantitative analysis of TNF-α, IL-1β, and IL-6 in BALF. The data represent mean ± SEM for groups of six rats (^##^ *P* < 0.01 vs OVXC group; ^*^ *P* < 0.05, ^**^ *P* < 0.01 vs OVXM group).

Endotoxin-induced proinflammatory cytokines such TNF-α, IL-1β, and IL-6 have been linked to the development of ALI [33,34]. In addition, the formation of ALI is caused by the activation of circulating neutrophils and their transmigration into the alveolar airspace. As a result, we looked at the expression of TNF-α, IL-1β, and IL-6 in BALF. The expression of TNF-α, IL-1β, and IL-6 were all considerably decreased by soy isoflavone, as predicted. Furthermore, the high-dose soy isoflavone had the same impact as the estradiol pre-treatment (Figure 3).

### 3.4 Soy isoflavone exhibits dose-dependent antioxidant effect to prevent LPS-induced ALI

**Figure 4:**
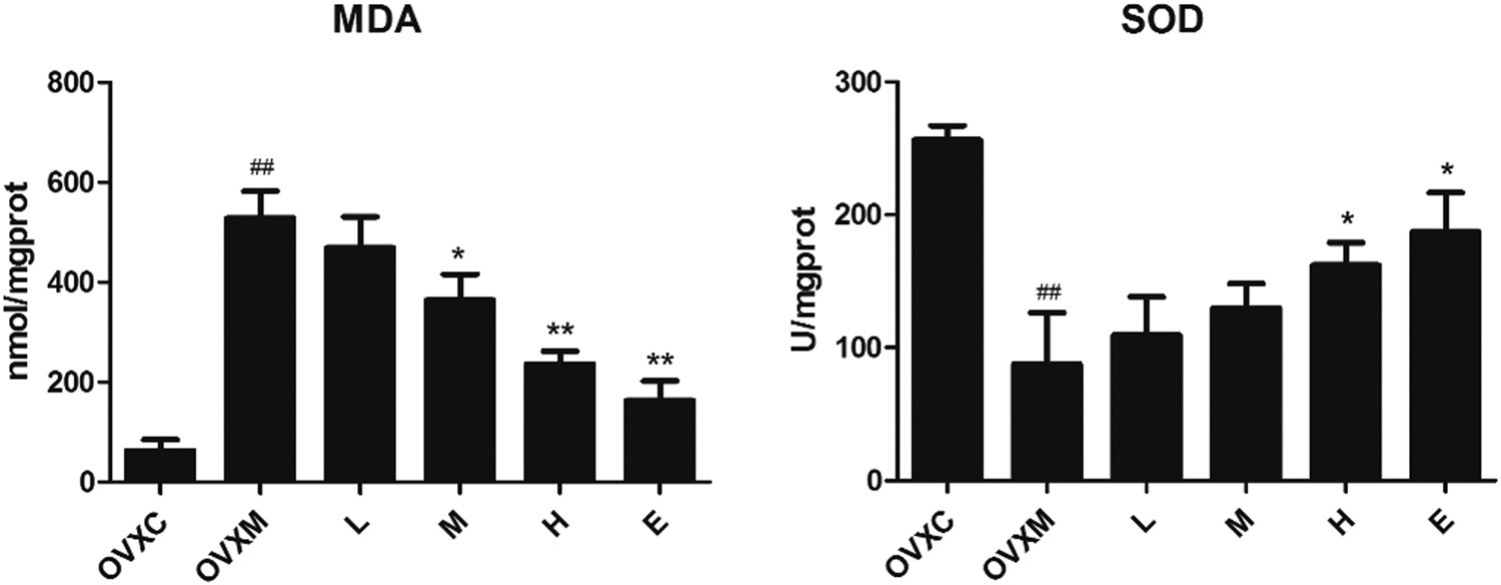
Soy isoflavone reduces MDA while increasing SOD in a dose-dependent manner. The quantitative analysis of MDA and SOD in lung tissues. Data represent mean ± SEM for groups of six rats (^##^ *P* < 0.01 vs OVXC group; ^*^ *P* < 0.05, ^**^ *P* < 0.01 vs OVXM group).

Lung tissues were assessed 6 h after LPS treatment to determine the effects of soy isoflavone on LPS-induced pulmonary oxidative stress. We discovered that treating rats with LPS caused substantial pulmonary oxidative stress by decreasing MDA levels while increasing SOD levels in lung tissues. Pre-treatment with soy isoflavone decreased LPS-induced MDA while raising lung tissues SOD levels in a dose-dependent manner in rats. Furthermore, the high-dose soy isoflavone had the same impact as the estradiol pre-treatment (Figure 4). These findings point out that soy isoflavone pre-treatment can treat LPS-induced ALI.

### 3.5 AQP1 and AOP5 mRNA and protein expression are increased by soy isoflavone

**Figure 5:**
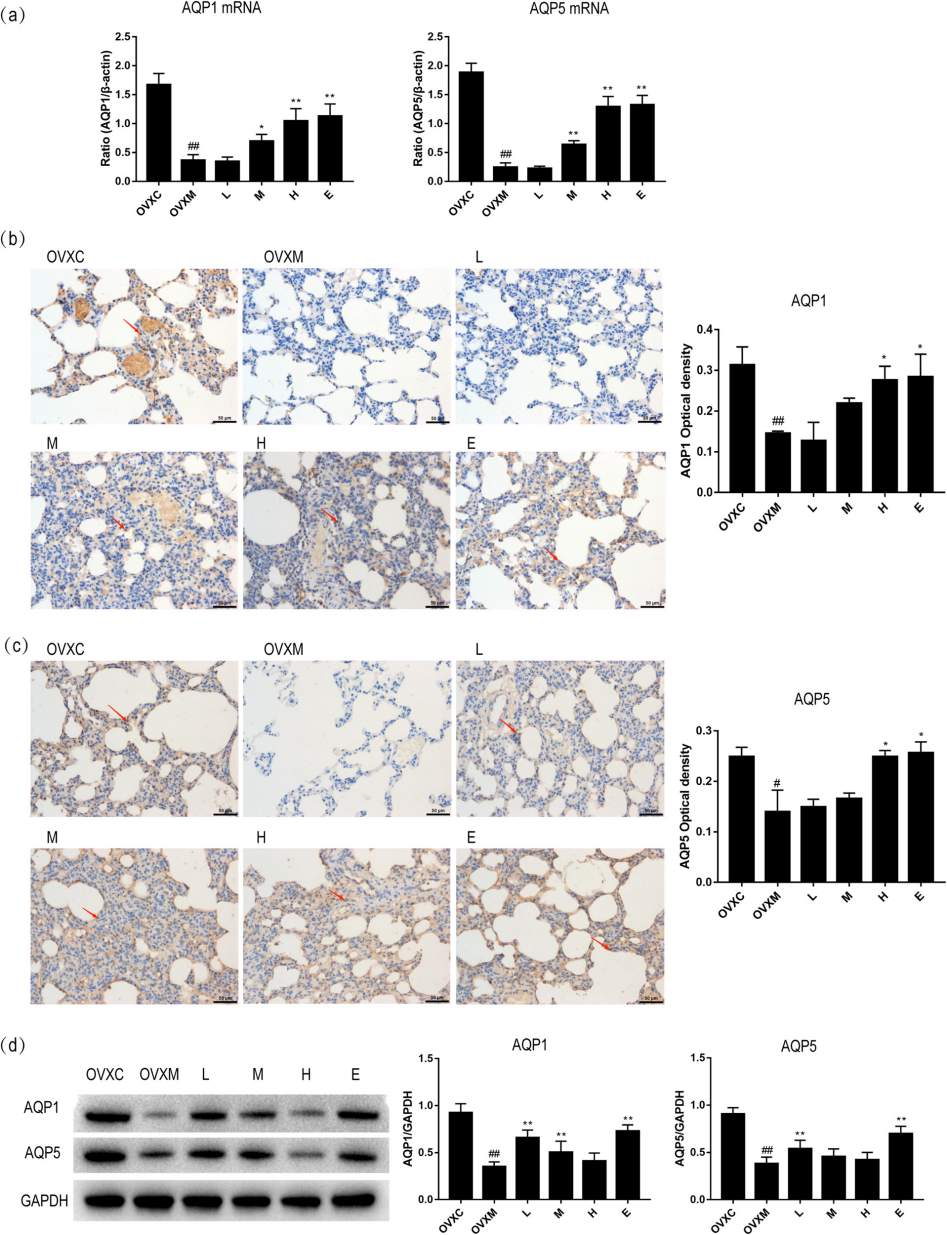
Soy isoflavone increases the mRNA and protein of AQP1 and AQP5 of lung tissues in a dose-dependent manner. (a) Quantitative analysis of AQP1 and AQP5 in lung tissues, *n* = 6. (b) Representative pictures and quantitative analysis of AQP1 in lung tissues by immunohistochemistry, *n* = 4, the red arrow shows the expression of AQP1 in lung tissue. (c) Representative pictures and quantitative analysis of AQP5 in lung tissues by immunohistochemistry, *n* = 4, the red arrow shows the expression of AQP5 in lung tissue. (d) Representative images and quantitative analysis of AQP1 and AQP5 in lung tissues by western blot, *n* = 6. Data represent mean ± SEM for groups of 4–6 rats, scale bar = 50 μm (^#^ *P* < 0.05, ^##^ *P* < 0.01 vs OVXC group; ^*^ *P* < 0.05, ^**^ *P* < 0.01 vs OVXM group).

According to studies, AQP1 and AQP5 are involved in the development of pulmonary edema caused by ALI [19]. Following that, we looked at the expression of AQP1 and AQP5 in lung tissues. We discovered that when rats were given LPS, the mRNA level of AQP1 and AQP5 were significantly decreased in lung tissues compared with OVXC group. Interestingly, pre-treatment with soy isoflavone up-regulated the mRNA expression of AQP1 and AQP5 in rats more than the OVXM group in a dose-dependent manner. Furthermore, the high-dose soy isoflavone had the same impact as the estradiol pre-treatment (Figure 5a). Moreover, we found that AQP1 and AQP5 protein expression was elevated dose-dependently in rats pre-treatment with soy isoflavone more than that in OVXM group by immunohistochemistry and western blot. Furthermore, the high-dose soy isoflavone had the same impact as the estradiol pre-treatment (Figure 5b). These findings point out that soy isoflavone pre-treatment can exhibit therapeutic effects in LPS-induced ALI.

## 4 Discussion

We employed soy isoflavone to treat ALI in rat model in this work. We discovered that soy isoflavone was protective in a dose-dependent manner and offered therapeutic promise for LPS-induced ALI. The results of a pre-treated trial with soy isoflavone showed that rats given LPS had severe ALI with lung edema, inflammation, and oxidative stress damage, which was inhibited by a pre-treated research with soy isoflavone. Importantly, we discovered that high-dose soy isoflavone had the same impact as pre-treatment with estrogen. As a consequence of the findings of this investigation, soy isoflavone is helpful and may have therapeutic potential for ALI.

Reduced inflammatory and erythrocyte infiltration in lung tissues can alleviate ALI mice considerably [35]. Soy isoflavone has anti-inflammatory and antioxidant properties, and is used to treat cancer [10], cardiovascular diseases [11], diabetes [12,13], and cerebrovascular disease [14]. In rats, soy isoflavone treatment can reduce alcoholic liver injury, ischemia/reperfusion injury, and radiation-induced lung injury [36,37], as well as radiation-induced lung injury in mice [38]. As a result, soy isoflavone may be beneficial to wounded tissues. We wanted to see if there was a new therapeutic effect of soy isoflavone in LPS-induced ALI. We pre-treated rats with soy isoflavone 14 days before administering LPS to examine the therapeutic impact of soy isoflavone on LPS-induced ALI. The therapeutic functions of soy isoflavone were obvious in rats after 6 h of LPS stimulation, with the therapeutic effects of soy isoflavone effectively attenuating pathological alterations in lung tissues (Figures 1 and 2). These findings clearly show that soy isoflavone has both preventative and therapeutic effects on LPS-induced ALI.

A range of respiratory inflammatory disorders, including acute respiratory distress syndrome, asthma, cystic fibrosis, and chronic obstructive pulmonary disease (COPD), have been related to the pathogenesis of redundant reactive oxygen species (ROS) [39]. ROS can cause inflammation in the airways and lungs by activating redox-sensitive transcription factors such activator protein 1 (AP1), hypoxia-inducible factor 1 (HIF-1) 1, and nuclear factor kappa-B (NF-κB) [40]. In ALI, oxidative stress is critical [41,42]. The current findings revealed that soy isoflavone not only had anti-inflammatory effect on lung injury, as evidenced by lower inflammatory cell infiltration, TNF-α, IL-1β, and IL-6 in BALF (Table 1, Figure 3), but also had an antioxidant impact on lung injuries, as evidenced by lower MDA levels and increased SOD levels (Figure 4).

AQP1 and AQP5 can attenuate lung edema by exerting antioxidant and anti-inflammatory functions in lung injury [18,20]. However, it is yet uncertain if soy isoflavones may protect against LPS-induced ALI by upregulating AQP1 and AQP5.

In this study, we proved that the AQP1 and AQP5 were decreased in the LPS-induced ALI model of rats. In rats, pre-treatment with soy isoflavones increased the expression of AQP1 and AQP5 in lung tissues in a dose-dependent manner (Figure 5). These findings imply that soy isoflavone has anti-inflammatory and antioxidant effects in LPS-induced ALI by inducing AQP1 and AOP5.

At present, many natural products have been studied to play a protective role through different mechanisms. Abdalla et al. found that safranal inhibits angiogenesis via targeting HIF-1α/VEGF machinery and exhibits anti-cancer property [43]. H_2_S displayed a hepatoprotective effect against cyclophosphamide-induced hepatotoxicity mediated by TLRs-JNK/NF-κB pathways to anti-inflammatory and antiapoptotic effects, reducing hepatic level TNF-α and caspase-3 expression [44]. Hawthorn herbal preparation from *Crataegus oxyacantha* attenuates carbon tetrachloride-induced hepatic fibrosis via modulating oxidative stress and inflammation in animals [45]. Similar to cyclophosphamide-induced liver injury, LPS-induced ALI also results in severe inflammation, oxidative stress, and apoptosis. A recent study found that isoflavones contributed to protecting from ALI via inhibiting toll-like receptor 4 (TLR4)/Myd88/NF-κB pathway and play anti-inflammation role [46]. Despite the fact that our findings imply that soy isoflavone via increasing AQP1 and AQP5 expression to reduce inflammation and oxidative stress, the exact mechanism needs to be researched further, especially the antioxidant. For example, NF-κB-mediated inflammation [35], coagulation/fibrinolysis system imbalance, cell apoptosis, autophagy and pyrosis of cells, ENaC, Na, K-ATPase, chloride ion channel, and other factors may influence pulmonary edema, and we should investigate them more.

## 5 Conclusion

Our findings reveal that soy isoflavone functions as anti-inflammatory and antioxidant through activating the AQP1 and AQP5 in LPS-induced ALI. Although these findings show soy isoflavones may protect against ALI by inducing AQP1 and AQP5, molecular alterations in ALI could nullify the effectiveness of such soy flavones. The molecular mechanism of natural plant-derived substances may be complex, and the present study only involves the change of AQP1 and AQP5 expression level. Further studies and study models are needed to demonstrate the therapeutic potential of soy flavones in ALI.
